# Effect of the physical rehabilitation program based on self-care ability in patients with acute ischemic stroke: a quasi-experimental study

**DOI:** 10.3389/fneur.2023.1181651

**Published:** 2023-06-09

**Authors:** Ying Li, Qian Wang, Xiao-Ling Liu, Rong Hui, Yin-Ping Zhang

**Affiliations:** ^1^Department of Nursing, Shaanxi Provincial People's Hospital, Xi'an, Shaanxi, China; ^2^Department of Urology, Shandong Provincial Hospital Affiliated to Shandong First Medical University, Jinan, Shandong, China; ^3^School of Nursing, Xi'an Jiaotong University Health Science Center, Xi'an, Shaanxi, China; ^4^Department of Neurology, Shaanxi Provincial People's Hospital, Xi'an, Shaanxi, China

**Keywords:** physical rehabilitation, acute ischemic stroke, myodynamia, self-care ability, quality of life

## Abstract

**Introduction:**

It is the most practical goal of limb rehabilitation for stroke patients to make the upper limb, trunk, and lower limb joints link together as a whole and restore the ability to self-care. However, many previous studies focused on the single joint or single muscle group movement of stroke patients and did not integrate self-care ability training into the whole process of rehabilitation, which lacks accuracy, integrity, and systematization.

**Methods:**

A quasi-experimental study was conducted in a tertiary hospital. Eligible patients were recruited according to the inclusion criteria and exclusion criteria and then divided into an experimental group (*n* = 80) and a control group (*n* = 80) by the medical district. The control group received the routine physical rehabilitation intervention. The experimental group adopted the physical rehabilitation program based on self-care ability led by the nurses specializing in stroke rehabilitation to carry out the multi-joint coordinated exercise based on the control group. The training time and frequency were the same in both groups (45 min per session, one session per day for three consecutive months). The primary outcome was myodynamia. Secondary outcomes were the modified Barthel Index (MBI) and Stroke Specific Quality of Life Scale (SS-QOL). The primary and secondary outcomes were assessed before the intervention and at 1 and 3 months of intervention. In this study, the TREND checklist was followed for non-randomized controlled trials.

**Results:**

A total of 160 participants completed the study. The physical rehabilitation program based on self-care ability was better than the routine rehabilitation program. With the prolongation of intervention time, all outcomes improved gradually in the experimental group (*P* < 0.05), and the myodynamia of lower limbs recovered faster than that of upper limbs. In the control group, the myodynamia of the affected limb was not significantly improved (*P* > 0.05), with only a small increase in MBI and SS-QOL scores (*P* < 0.05).

**Conclusion:**

The physical rehabilitation program based on self-care ability after stroke was beneficial for acute ischemic stroke patients and improved the patient's myodynamia, quality of life, and self-care ability within the third month.

## 1. Introduction

Stroke has become the leading cause of death and disability worldwide (1, 2). Acute ischemic stroke (AIS) accounts for about 70%, and more than one-third of AIS patients die or become disabled within 3 months or 1 year (3, 4). China has a high number of stroke patients in the world. There are ~2.4 million new stroke cases in China every year, and ~70%−80% of AIS patients are accompanied by a disability and cannot live independently (5). Limb dysfunction is the most common dysfunction in stroke patients. Patients usually have different degrees of damage to the motor conduction pathway and motor cells, resulting in the weakening of the ability to actively control a limb, myodynamia, and muscle tension (6). AIS patients need help from others in eating, dressing, and bathing (7). In addition, they suffer from a decline in self-confidence and self-efficacy due to the diseased limb dysfunction, which seriously affects their self-care ability and quality of life (8).

Self-care ability assessment is not only the basis of treatment, nursing, and rehabilitation but also one of the most important indicators to evaluate the effect of rehabilitation for AIS patients (9, 10). Studies have shown that improving patients' self-care ability is the most practical goal of stroke rehabilitation, and the improvement of AIS patients' quality of life has been related to self-care ability (11). Therefore, improving self-care ability is critical for AIS patients. At present, a large number of studies have used self-care ability as outcomes, but there have been few rehabilitation programs based on self-care ability, and many rehabilitation programs lack a clear standard or basis. There are many scales to judge the self-care ability of AIS patients, but the MBI scale is a better choice in terms of the difficulty of operation, the completeness of items, and the standardization of the scale (12). The MBI has been globally recognized as the most commonly used scale to evaluate self-care ability, which is simple, reliable, and sensitive. It comprises 10 items including eating, dressing, bathing, and walking and divides the self-care ability of patients into five levels: complete dependence, severe dependence, moderate dependence, mild dependence, and basic self-care life. It is beneficial for doctors and nurses to accurately evaluate patients and develop an accurate physical rehabilitation program for AIS patients.

The improvement of motor function and self-care ability in AIS patients is a slow and long-term process (13). However, with the increase in the number of patients, the hospitalization period of AIS patients in hospitals has gradually become shorter, and the turnover rate has increased. Many countries, especially developing countries, have had a shortage of rehabilitation professionals and qualified rehabilitation institutions (14, 15). In addition, due to repeated COVID-19 in recent years, most patients chose to recover at home after being discharged from the hospital (16, 17). During hospitalization, the patient is mainly treated by clinical medical methods in the Department of Neurology such as drug therapy. Routine rehabilitation training mainly includes active and passive joint training in bed with one joint or one muscle group and walking training, which is associated with a higher rate of relapse and loss to follow-up rate. This program involves less training, separates the upper and lower limbs of AIS patients, and ignores the differences in patients. Long-term training of AIS patients with single joint or single muscle group after discharge is adverse to their physical rehabilitation.

The physical rehabilitation training of AIS patients is a gradual and complex process, requiring the coordination of multiple joints and muscle groups (18, 19). Moreover, previous physical rehabilitation programs for AIS patients were relatively simple and lacked systematicness and accuracy. It was difficult to achieve continuous rehabilitation from admission to discharge with the strength of rehabilitation therapists alone. Therefore, it is an urgent problem to provide a systematic, accurate, and continuous physical rehabilitation strategy for stroke patients.

Overall, focusing on the five levels of self-care ability, this study constructed and implemented a physical rehabilitation program based on the self-care ability of AIS patients and carried out a 3-month intervention. The starting point of rehabilitation training was determined by rehabilitation therapists and neurology physicians according to the patient's self-care ability. The continuous rehabilitation plan, which included admission and discharge, was implemented by nurses specializing in stroke rehabilitation. This study developed a systematic, accurate, and continuous limb rehabilitation program for AIS patients, aiming to improve the patient's myodynamia, self-care ability, and quality of life and provide a reference for the rehabilitation of stroke patients.

## 2. Materials and methods

### 2.1. Participants

Patients were included if they were (1) diagnosed with AIS according to the Chinese guidelines for the diagnosis and treatment of AIS 2018 (20); (2) aged between 35 years and 75 years; (3) the first to have AIS; (4) stable, conscious hemiplegic patients; and (5) informed and agreed to participate in the experiment.

Patients were excluded if they (1) had evidence of intracranial hemorrhage or other cerebral diseases (e.g., vascular malformation, tumor, abscess, or multiple sclerosis); (2) had severe complications such as liver or renal dysfunction; (3) had evidence of massive ischemic stroke (more than one lobe of brain or over one-third of blood supply area of a middle cerebral artery) (21); (4) were legally disabled (e.g., were blind, deaf, or unable to speak; had a mental impairment or mental disorders) or had other physical disabilities that affected the evaluation of neurological impairment; (5) had experienced a hemorrhagic tendency or severe bleeding within the last 3 months; (6) were currently pregnant or lactating; (7) were participating in another clinical study or had participated in other studies in the last 3 months; and (8) exhibited any condition that in the view of the investigator would prevent study eligibility.

This study was approved by the Medical Ethics Committee of Shaanxi Provincial People's Hospital (the approval number was SPPH-LLBG-17-3.2). All the participants provided written informed consent.

### 2.2. Physical therapists

All the stroke physical rehabilitation therapists involved in this study obtained health professional and technical certificates approved and issued by the National Health and Family Planning Commission and the Ministry of Human Resources and Social Security of the People's Republic of China. They have been engaged in stroke limb rehabilitation for more than 5 years.

### 2.3. Design

This was a quasi-experimental study conducted in the neurology ward of a tertiary hospital in China. Given the ethical requirements and the nature of the intervention, the Department of Neurology was divided into two wards, each of which admitted patients who were homogeneous in terms of age, gender, type of disease, and treatment. We set one ward as a control group and the other as a experimental group. We assigned the patients to either the control or the experimental group using a random number table generated by computer and assigned to two groups in a 1:1 ratio by concealed sequentially numbered envelopes. In addition, participants were blinded to the grouping and intervention. A total of 160 participants were recruited and divided into the experimental group (*n* = 80) and control group (*n* = 80). The purpose of this study was to compare the effects of physical rehabilitation programs based on self-care ability led by nurses specializing in stroke rehabilitation with routine physical rehabilitation intervention. All participants were informed of the purpose of the study and agreed to participate in the study. The TREND checklist was followed for non-randomized controlled trials (see Supplementary).

### 2.4. Interventions

#### 2.4.1. Experimental group

According to the patient's level of self-care ability, members of the multidisciplinary rehabilitation team, including doctors, rehabilitation therapists, and nurses specializing in stroke rehabilitation, developed a physical rehabilitation plan for AIS patients (22) and conducted a 3-month study. According to the MBI score, the self-care ability of patients was divided into five levels in 20-point increments: complete dependence, severe dependence, moderate dependence, mild dependence, and basic self-care of life.

(1) During the hospitalization, rehabilitation therapists and doctors assessed the patient's condition, determined the level of self-care ability, and the starting point of rehabilitation according to the patient's MBI score and actual physical condition. Twenty-four hours after the patient's vital signs were stabilized, the rehabilitation training plan was implemented by the nurses specializing in stroke rehabilitation. (2) The WeChat group of physical rehabilitation for patients in the experimental group was established, and the group members included members of the multidisciplinary rehabilitation team, patients, and their caregivers. Nurses specialized in stroke rehabilitation pushed videos of the physical rehabilitation training to the group. (3) Before discharge, patients were informed to do physical rehabilitation training at home every day after discharge, and caregivers were informed to record the process of the patient's physical rehabilitation training in the form of video and feedback in the WeChat group every 2 weeks. (4) The patients entered the WeChat group upon discharge, and nurses specializing in stroke rehabilitation again explained and demonstrated to them and their caregivers the movement essentials and precautions of the rehabilitation training. (5) After discharge, the caregivers filmed the process of the patients' physical rehabilitation training at home in a video and provided feedback in the WeChat group once every 2 weeks. In the WeChat group, doctors and nurses specializing in stroke rehabilitation and rehabilitation therapists continued to follow up on the patient's training process and provided targeted guidance for the content of the feedback. For patients who did not provide feedback in time, nurses specializing in stroke rehabilitation reminded and urged patients to carry out physical rehabilitation at home via WeChat, and guided patients online to carry out physical rehabilitation training.

In order to improve the rehabilitation compliance of patients after discharge, the following measures were mainly adopted in this study. First of all, it not only provided patients with professional guidance on physical rehabilitation but also regularly arranged training for caregivers. During the period of hospitalization, the nurses specializing in stroke rehabilitation carried out rehabilitation training education for the caregivers in the teaching classroom on Monday, Wednesday, and Friday. Second, when the nurse implemented rehabilitation training for patients in the ward, she also explained training methods and matters needing attention for caregivers. After assisting the patients with rehabilitation training, the nurse instructed the patients to repeat the same training. Before the patients were discharged, the nurse explained and demonstrated the method of physical rehabilitation training for the caregivers again. When patients were discharged from the hospital, the Department of Neurology arranged online rehabilitation courses free of charge for caregivers every 2 weeks and pushed rehabilitation training videos in the WeChat group. The nurses specializing in stroke rehabilitation urged and guided caregivers to consolidate rehabilitation actions when urging patients to provide feedback on WeChat every 2 weeks. In addition, different rewards were given for the consecutive times of feedback.

#### 2.4.2. Control group

Routine drugs and rehabilitation therapy were given to patients in the Department of Neurology every day. Within 24 h after the patient's condition stabilized, nurses in the Department of Neurology provided rehabilitation training guidance for them and their caregivers, mainly with passive and active joint activity training in bed with the single joint or single muscle group and walking training assisted by walking AIDS. After discharge, patients were also urged to perform routine limb rehabilitation training. The training time, intensity, frequency, caregiver training, and compliance improvement methods of patients in the control group were the same as those in the experimental group.

#### 2.4.3. Outcome measures

The primary outcomes were the six levels of the myodynamia assessment method commonly used in clinical practice. Level 0 myodynamia is complete paralysis of the limb, and muscle contractions cannot be seen or measured by the eye. The myodynamia of level 1 is that the muscle contractions can be seen by the eye, but no movement can be formed. Level 2 myodynamia is the ability of the limbs to move in parallel on the bed, but it cannot resist gravity and be lifted off the bed surface. Level 3 myodynamia is that the limbs can resist gravity and lift off the bed surface but cannot resist resistance. Level 4 myodynamia is the ability of the limb to resist only partial resistance. Level 5 myodynamia indicates that the patient has normal myodynamia.

The secondary outcomes were the MBI and SS-QOL scales. The self-care ability was evaluated by the MBI, comprising 10 items including eating, dressing, bathing, and grooming (12), with a range of 0 to 100 points. The higher the score, the better the patient's self-care ability. The quality of life was assessed by the Chinese version of the SS-QOL (23), which includes 12 dimensions and 49 items with a total score of 49–245. The higher the score, the better the quality of life.

Myodynamia, MBI, and SS-QOL were assessed at baseline (t0), 1 month (t1), and 3 months (t2) of intervention. We also recorded sociodemographic and clinical data. Patients directly reported any unintentional injuries and serious adverse events during hospitalization or follow-up.

### 2.5. Statistical analysis

Descriptive statistics were applied, with results provided as numbers and percentages for categorical variables and means with SD for continuous variables. When the data conform to the normal distribution, between-group data were compared using the independent *t*-test, chi-square, or Fisher's exact test. A non-parametric test was used when the data did not conform to normal distribution. The Mann–Whitney *U*-test in rank-sum test was used to compare grade data. The repeated measurement data of outcomes were analyzed using GEE or analysis of variance of repeated measurement data. Data analysis was performed using SPSS V.25.0 (IBM Corporation, US).

## 3. Results

### 3.1. Participants' characteristics

According to our sample size, 178 patients with stroke were recruited from our hospital for a period of 3 months. Among the 178 participants, the data at 3 months of intervention were available for 160, including five participants who had died, ten who were lost to follow-up, and three who had missing data. Finally, 80 patients in each group were determined. A total of 160 AIS patients participated in all outcome assessments. Characteristics of the participants were similar between groups, and outcome measures between the two groups showed no significant differences (*P* > 0.05). These results suggested that the groups were comparable at baseline (Table 1).

**Table 1:** Baseline demographic and clinical characteristics between groups *n* (%).

**Variables**	**Control group**	**Experimental group**	**χ^2^*/t* value**	***P*-value**
Gender (example)			χ^2^ = 0.271	0.603
Man	58 (72.50)	55 (68.75)		
Woman	22 (27.50)	25 (31.25)		
Age (years)	58.49 ± 10.36	59.76 ± 11.90	*t* = −0.720	0.471
Education level			χ^2^ =1.279	0.528
Primary school and below	22 (27.50)	26 (32.50)		
Junior high school	22 (27.50)	25 (31.25)		
High school or above	36 (45.00)	29 (36.25)		
Primary caregiver			χ^2^ = 4.756	0.191
Spouse-dominated	47 (58.75)	41 (51.25)		
Children-oriented	13 (16.25)	14 (17.50)		
Spouses and children	16 (20.00)	13 (16.25)		
Others	4 (5.00)	12 (15.00)		
Per capita monthly income of families (yuan)			χ^2^ = 1.285	0.739
< 500	13 (16.25)	11 (13.75)		
500–1,000	10 (12.50)	15 (18.75)		
1,001–3,000	28 (35.00)	26 (32.50)		
>3,000	29 (36.25)	28 (35.00)		
Family history of stroke			χ^2^ = 1.731	0.188
No	47 (58.75)	55 (68.75)		
Yes	33 (41.25)	25 (31.25)		
Smoking			χ^2^ = 0.227	0.634
No	45 (56.25)	42 (52.50)		
Yes	35 (43.75)	38 (47.50)		
Drinking alcohol			χ^2^ = 0.440	0.507
No	50 (62.50)	54 (67.50)		
Yes	30 (37.50)	26 (32.50)		
Hemiplegic side			χ^2^ = 0.400	0.527
Right	41(51.25)	37 (46.25)		
Left	39 (48.75)	43 (53.75)		
NIHSS scores (score)	12.11 ± 3.34	12.21 ± 3.60	*t* = −0.183	0.855

### 3.2. The grade comparison of myodynamia

The Wilcoxon rank-sum test was used to compare the grade comparison of myodynamia. There was no significant difference in myodynamia of affected limbs between the two groups at t0 (upper limb: *Z* = −4.494, *P* = 0.621; lower limb: *Z* = −0.158, *P* = 0.871) and t1 (upper limb: *Z* = −0.163, *P* = 0.870; lower limb: *Z* = −0.841, *P* = 0.400). However, the myodynamia of affected limbs in the experimental group was better than that in the control group at t2, and the difference between the groups was statistically significant (*P* < 0.01). In the experimental group, only the difference in myodynamia at 3-time points before and after the intervention was statistically significant (*P* < 0.01). By pairwise comparison, myodynamia was gradually improved with the extension of the intervention time (all adjusted *P* < 0.05; Tables 2, 3).

**Table 2:** Grade of myodynamia of affected upper limb in each group *n* (%).

**Time**	**Myodynamia**	**Control group**	**Experimental group**	***Z*-value^a^**	***P*-value**
Baseline	Level 0	10 (12.50)	14 (17.50)	−4.494	0.621
	Level 1	15 (18.75)	17 (21.25)		
	Level 2	21 (26.25)	15 (18.75)		
	Level 3	25 (31.25)	24 (30.00)		
	Level 4	9 (11.25)	10 (12.50)		
1 month of intervention	Level 0	3 (3.75)	4 (5.00)	−0.163	0.870
	Level 1	23 (28.75)	23 (28.75)		
	Level 2	19 (23.75)	15 (18.75)		
	Level 3	26 (32.50)	28 (35.00)		
	Level 4	8 (10.00)	10 (12.50)		
	Level 5	1 (1.25)	0 (0.00)		
3 months of intervention	Level 0	2 (2.50)	0 (0.00)	−3.563	< 0.001
	Level 1	16 (20.00)	8 (10.00)		
	Level 2	29 (36.25)	22 (27.50)		
	Level 3	25 (31.25)	23 (28.75)		
	Level 4	7 (8.75)	26 (32.50)		
	Level 5	1 (1.25)	1 (1.25)		
*H*-value^b^		0.498	20.647		
*P-*value		0.780	< 0.001		

a Mann–Whitney U-test for two independent samples.

b Kruskal–Wallis H-test for three independent samples.

**Table 3:** Grade of myodynamia of affected lower limb in each group n(%).

**Time**	**Myodynamia**	**Control group**	**Experimental group**	***Z*-value^a^**	***P*-value**
Baseline	Level 0	6 (7.50)	3 (3.75)	−0.158	0.874
	Level 1	15 (18.75)	17 (21.25)		
	Level 2	20 (25.00)	19 (23.75)		
	Level 3	26 (32.50)	35 (43.75)		
	Level 4	12 (15.00)	6 (7.50)		
	Level 5	1 (1.25)	0 (0.00)		
1 month of intervention	Level 0	5 (6.25)	1 (1.25)	−0.841	0.400
	Level 1	15 (18.75)	16 (20.00)		
	Level 2	25 (31.25)	21 (26.25)		
	Level 3	25 (31.25)	34 (42.50)		
	Level 4	9 (11.25)	8 (10.00)		
	Level 5	1 (1.25)	0 (0.00)		
3 months of intervention	Level 0	4 (5.00)	0 (0.00)	−3.47	0.001
	Level 1	20 (25.00)	5 (6.25)		
	Level 2	24 (30.00)	27 (33.75)		
	Level 3	23 (28.75)	30 (37.50)		
	Level 4	8 (10.00)	18 (22.50)		
	Level 5	1 (1.25)	0 (0.00)		
*H*-value^b^		0.939	40.663		
*P-*value		0.625	< 0.001		

a Mann–Whitney U-test for two independent samples.

b Kruskal–Wallis H-test for three independent samples.

### 3.3. The scores of MBI

The scores of MBI in the two groups were non-normal distributions and were generally normal distributions after rank transformation. The following were the results after data rank conversion. GEE analysis showed that there was an interaction effect between time and group. The difference in MBI score between the experimental group and the control group was not statistically significant at t0 (Wald χ^2^ = 0.667, *P* = 0.411). The MBI score of patients in the control group was only significantly higher at t2 than that at t0 (Wald χ^2^ = 204.967, *P* < 0.01). However, the MBI scores in the experimental group were higher than those in the control group (*P* < 0.01; Table 4).

**Table 4:** MBI scores for each group and the results of GEE analysis with interaction.

**Parameter**	**Exp (*B*)**	**SE**	**95% Wald confidence interval for Exp (** ***B*** **)**		**Wald Chi-square**	*P-*value^*^
			**Lower**	**Upper**		
(Intercept)	−0.398	0.0909	−0.576	−0.220	19.172	< 0.001
[group = 1]	0.126	0.1526	−0.174	0.425	0.677	0.411
[group = 0]	0^a^					
[time = 2]	0.773	0.0540	0.667	0.879	204.967	< 0.001
[time = 1]	0.018	0.0100	−0.002	0.038	3.181	0.074
[time = 0]	0^a^					
[group = 1] ^*^ [time = 2]	0.273	0.0799	0.116	0.430	11.675	0.001
[group = 1] ^*^ [time = 1]	0.159	0.0353	0.090	0.228	20.270	< 0.001
[group = 1] ^*^ [time = 0]	0^a^					

* Results of generalized estimating equation (GEE) analysis for the group by time effect across all timepoints. group = 0: Control group, group = 1: experimental group. time = 0: baseline; time = 1: 1 month of intervention; time = 2: 3 months of intervention.

MBI, modified Barthel index.

### 3.4. The scores of SS-QOL

Repeated measurement analysis of variance by Greenhouse–Geisser corrected showed that the SS-QOL scores of the experimental group were higher than those of the control group after intervention (*P* < 0.01), and the SS-QOL scores of the two groups at t2 were higher than those that at t1 (*P* < 0.01). Moreover, there was an interaction effect between time and group (*F* = 146.294, *P* < 0.01). With the extension of intervention time, the SS-QOL scores of the two groups gradually increased, and the increased range of the scores of the experimental group was greater than that of the control group (Table 5).

**Table 5:** SS-QOL scores for each group and results of repeated measures analysis of variance.

	**Baseline (t0)**	**1 month (t1)**	**3 months (t2)**	**Time effect**		**Inter-group effect**		**Group** × time effect^*^	
				***F*** **-value**	***P-*** **value**	***F*** **-value**	***P*** **-value**	***F*** **-value**	***P-*** **value**
Control group (*n* = 80)	117.59 ± 17.99	120.10 ± 18.11	121.89 ± 16.89	35.602	< 0.01	34.401^a^	< 0.01	146.294	< 0.01
Experimental group (*n* = 80)	119.94 ± 22.78	130.83 ± 22.30	137.46 ± 21.70	433.476	< 0.01	75.636^b^	< 0.01		
*t* value	−0.724	−3.339	−5.066						
*P*-value	0.470	0.001	< 0.01						

Outcome measures presented as mean ± SD.

* Interaction results of time and group in repeated measurement analysis of variance.

a One month of intervention.

b Three months of intervention.

SS-QOL, stroke-specific quality of life scale.

## 4. Discussion

This quasi-experimental study found that a physical rehabilitation program based on self-care ability was superior to a routine physical program. In the experimental group, myodynamia, self-care ability, and quality of life of patients were significantly improved, and myodynamia of the affected lower limb recovered faster than that of the upper limb.

Dyskinesia in stroke patients was mainly manifested in abnormal movement patterns (24). Spasticity was the main factor of motion control disorder and abnormal motion patterns in stroke patients (25). Long-term spasticity can make pathological changes in muscle and bone and even produce various complications. Research showed that a daily range of motion exercise was the most basic part of dealing with spasms (26). The antispasmodic training and resistance exercise in the experimental group were found to be able to inhibit α motor neurons and brainstem reticular structure (25).

Myodynamia of lower limbs recovered faster than that of upper limbs, which may be related to the following factors. The lower limb sensorimotor area of the brain received a dual blood supply from the middle cerebral artery and the anterior cerebral artery, while the upper limb sensorimotor area received a single blood supply (27). The upper limb was responsible for finer movements, such as holding, grasping, and kneading. The sensorimotor area in the brain of upper limbs was more widely distributed, and patients have more damage after stroke, so the recovery of upper limb function was slower. The upper limb on the healthy side can replace the affected upper limb to complete some basic movements (28), so some patients did not pay much attention to the upper limb. As long as the healthy hand could be used, some patients did not even use the affected hand, so it was more difficult to recover the upper limb. Walking, climbing stairs, and other activities needed the coordination and cooperation of the affected and healthy lower limbs, so the affected lower limbs had more opportunities to exercise and were easier to recover. Therefore, the physical rehabilitation program based on self-care ability enabled the patients' upper and lower limbs to coordinate and move together, with great attention to ADL training. Except for the patients who were completely unable to take care of themselves, the other patients were given ADL training so that the AIS patients could use their affected side upper limbs for mobility to the greatest extent, such as washing their face, drinking water, and eating. In addition, the experimental group also implemented electromyographic biofeedback therapy (EMG-BFB) treatment for stroke patients, which can replace proprioceptive receptors, promote the regeneration of nerve fibers in AIS patients, and improve the control ability of the affected limb (29, 30). This is similar to the results of Kim JH's study (31).

The quality of life of AIS patients depends on the function of their affected limbs and their self-care ability (32). It has been found that physical rehabilitation training led by nurses specializing in stroke rehabilitation not only improved the enthusiasm and self-efficacy of rehabilitation training but also reduced the psychological burden of patients and their caregivers (33, 34). It is very beneficial to improve the patient's self-care ability and quality of life. Compared with doctors and rehabilitation therapists, nurses were found to have more contact with patients and were more able to integrate patients' daily training into patients' daily life (35) so as to improve patients' training compliance. In addition, the patients in the experimental group have been receiving systematic and continuous rehabilitation training, regular follow-up, and supervision of nurses specializing in stroke rehabilitation. Through rehabilitation training, AIS patients gradually saw and felt that their limb function was getting better, which further enhanced their confidence and self-efficacy in physical rehabilitation.

Therefore, it was necessary to construct a physical rehabilitation program for AIS patients based on self-care ability. We should not only attach great importance to the rehabilitation of stroke patients but also provide more systematic and accurate physical rehabilitation strategies. In addition, the hospital should also reasonably allocate medical resources and strengthen the training of nurses specializing in stroke rehabilitation so as to improve patients' self-care ability and quality of life.

## 5. Limitations and perspectives

This study had several limitations. First, it is a single-center study with a limited sample size, and the applicability and generalizability of the findings will be limited. Further multicenter studies with larger sample sizes are needed to support our conclusions. Second, considering the cooperation and compliance of patients at follow-up, our study only evaluated the effects of physical rehabilitation programs on myodynamia, quality of life, and self-care ability. Third, most of our data were collected from rehabilitation scales and lacked sufficient quantitative and objective measures, which may introduce some bias to the evaluation. Finally, this rehabilitation method can cause any potential adverse reactions such as falls, bed falls, or pressure sores. However, before the implementation of the study plan, we have developed the relevant contingency plans. During the implementation process, we also modified and improved the contingency plan continuously according to the actual situation. In future studies, we will apply objective measures, increase interventions, and increase the sample size to carry out multicenter clinical trials to identify risk factors for AIS patients, as well as improve and refine the follow-up system, enhance communication with patients, and increase outcome indicators.

## 6. Conclusion

The physical rehabilitation program for AIS patients based on self-care ability may be beneficial, as it improved the patient's myodynamia, quality of life, and self-care ability within the third month. Due to the limited time, this study was only conducted in a tertiary hospital in China for 3 months. It is necessary to conduct independently replicated studies with large samples to validate our findings.

## Data availability statement

The original contributions presented in the study are included in the article/Supplementary material, further inquiries can be directed to the corresponding authors.

## Ethics statement

The studies involving human participants were reviewed and approved by Medical Ethics Committee of Shaanxi Provincial People's Hospital. The patients/participants provided their written informed consent to participate in this study.

## Author contributions

RH and Y-PZ designed the study, oversaw data safety and monitoring, and revised the manuscript. YL performed the analysis and drafted the manuscript. QW and X-LL were site principal investigators and oversaw site recruitment and site data collection. All authors contributed to the manuscript's initial draft, read, and approved the final manuscript.
